# Mitochondria Synthesize Melatonin to Ameliorate Its Function and Improve Mice Oocyte’s Quality under *in Vitro* Conditions

**DOI:** 10.3390/ijms17060939

**Published:** 2016-06-14

**Authors:** Changjiu He, Jing Wang, Zhenzhen Zhang, Minghui Yang, Yu Li, Xiuzhi Tian, Teng Ma, Jingli Tao, Kuanfeng Zhu, Yukun Song, Pengyun Ji, Guoshi Liu

**Affiliations:** National Engineering Laboratory for Animal Breeding, Key Laboratory of Animal Genetics and Breeding of the Ministry of Agriculture, Beijing Key Laboratory for Animal Genetic Improvement, College of Animal Science and Technology, China Agricultural University, Beijing 100193, China; chungjoy@cau.edu.cn (C.H.); caylajingjing@gmail.com (J.W.); taoyaerxl@gmail.com (Z.Z.); caylahuihui@gmail.com (M.Y.); caylapangpang@gmail.com (Y.L.); future830502@gmail.com (X.T.); mateng7777@gmail.com (T.M.); tjl11325@gmail.com (J.T.); suxingdemogu@gmail.com (K.Z.); songyukun@cau.edu.cn (Y.S.); jipengyun1989@gmail.com (P.J.)

**Keywords:** antioxidant, melatonin, mitochondria, oocyte, *SNAT*

## Abstract

The physiology of oocyte *in vitro* maturation remains elusive. Generally, the oocytes have a very low maturation rate under *in vitro* conditions. In the current study, we found that melatonin promotes the maturation of oocytes in which mitochondria play a pivotal role. It was identified that; (1) mitochondria are the major sites for melatonin synthesis in oocytes and they synthesize large amounts of melatonin during their maturation; (2) melatonin improves mitochondrial function by increased mtDNA copy, mitochondrial membrane potential (ΔΨ_m_) and mitochondrial distribution and ATP production in oocytes; (3) the meiotic spindle assembly is enhanced; (4) melatonin reduces ROS production and inhibits 8-oxodG formation, thereby protecting potential DNA mutation from oxidative damage. As a result, melatonin improves the quality of oocytes, significantly accelerates the developmental ability of IVF embryo. The results provide novel knowledge on the physiology of oocyte’s maturation, especially under *in vitro* conditions.

## 1. Introduction

Female fertility is mainly determined by the quality of oocytes which are susceptible to multifaceted factors such as environment stressors, availabilities of essential nutrients, *etc*. [1,2]. Maintaining a normal maturation process is critical for obtaining oocytes of high-quality. Oocyte maturation is a highly coordinated process involving both nuclear and cytoplasmic events, which are composed of both nuclear and cytoplasmic maturations [3,4]. These events include nuclear membrane breakdown and dissolution (GVBD), spindle assembly, epigenetic programming, mitochondrial accumulation and distribution, and reactive oxygen species (ROS) reduction [2]. A spectrum of genes also selectively participate in the process of oocyte maturation. The representative genes include EGF-like protein that mediates LH signaling [5], *GDF9* and *BMP15* which directly involve energy metabolism [6,7], *NPPC/NPR2* signaling which maintain meiosis arrest [8,9], Mos/MAPK-signal cascades and CyclinA_2_ which involves in meiotic progression and cell cycle control [10]. A newly-found oocyte-derived protein, meiosis arrest female1 (*MARF1*), has an important function in controlling meiosis, cytoplasmic development, and maintenance of genomic integrity [11,12]. Notably, the processes of both oocyte maturation and ovulation are usually comparable to an inflammatory response which is accompanied with large amounts ROS generation [13,14]. The excessive ROS, if not properly detoxified, would lead to a poor quality of the oocytes under both *in vivo* and *in vitro* conditions [15]. Oocytes are more resistant to oxidative stress during ovulation under the *in vivo* conditions. This may be attributed to the relatively high levels of antioxidants existing in the follicular fluid [16,17]. They serve as a protective means against ovary oxidative stress. 

Melatonin (*N*-acetyl-5-methoxytryptamine) is a derivative of tryptophan. This molecule is a naturally occurring potent free radical scavenger and a broad-spectrum antioxidant [18]. As a result, the use of melatonin to prevent oxidative stress in cell culture or animal studies has been extensively reported [19,20]. In addition to its antioxidant property, melatonin also plays an important role in animal reproduction. In rat, melatonin lowers estradiol level while it enhances progesterone production during early gestation. In mice, melatonin involves pregnant regulation and promotes embryo implantation [21,22]. The circadian variations of melatonin in the maternal circulation may regulate the timing of parturition [23]. Melatonin also affects ovarian function. A relatively high level of melatonin was found in human preovulatory follicular fluid [24,25]. This molecule was found to alleviate goat granulosa cells from the harmful effects of heat stress [26]. The actions of melatonin in physiological and pathological processes are usually mediated by its receptors, such as membrane receptors *MT1* and *MT2* [27]. These receptors were also expressed in the human granulosa cells and enhanced progesterone production [28,29]. The *SNAT* gene (encoding melatonin synthase, also named *AANAT*) expression and melatonin increases progressively in oocytes during follicular development [30]. However, the organelle synthesizing melatonin in oocytes has not been clarified and the actions of melatonin on ovulation are also not fully understood. 

In the current research, the subcellular localization of melatonin synthesis in oocyte was investigated. To clarify the mechanisms of melatonin on quality of oocyte and its maturation, the mitochondrial function, energy states, the spindle assembly, the ROS production, DNA oxidative damage and cellular apoptosis of oocytes have been systematically studied. 

## 2. Results

### 2.1. Oocytes Synthesize Melatonin during Maturation

**Figure 1:**
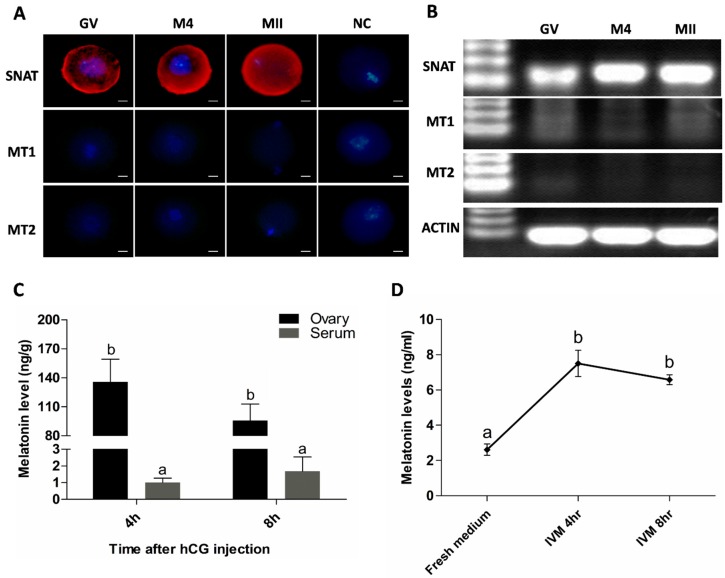
Oocyte synthesis melatonin during its maturation. (**A**) Assayed with immunofluorescence. Red was *SNAT* staining. Scale bar = 20 μm; (**B**) Assayed with RT-PCR. GV: Germinal Vesicle Stage, M4: 4 h after hCG injection, MII; Metaphase II of Meiosis, NC: Negative Control; (**C**) Melatonin levels in ovary homogenate and serum of the animals after hCG injection (mean ± SEM); (**D**) Melatonin levels in IVM medium during oocyte culture (mean ± SEM); the different superscript letters (a–b) represent a significant difference (*p* < 0.05).

It was observed that *SNAT* existed persistently at all stages of oocyte’s maturation. The proteins of both *MT1* and *MT2*, however, were undetectable in oocytes at every stage assayed with immunofluorescence (Figure 1A) or RT-PCR (Figure 1B). Melatonin was detected in the ovary and serum in the animals during the period of oocyte’s maturation. Melatonin levels in ovary at 4 and 8 h after hCG treatment were significantly higher than those in their contemporaneous sera (135.7 ± 23.51 *versus* 1.01 ± 0.260 and 95.7 ± 17.09 *versus* 1.69 ± 0.850 ng/g, respectively, *p* < 0.01) (Figure 1C). Meanwhile, melatonin was also detected in the oocyte’s cultured medium at 4 or 8 h, which was significantly higher than that in fresh medium (1.50 ± 0.150, 1.32 ± 0.056 *versus* 0.52 ± 0.066 ng/mL, respectively, *p* < 0.01) (Figure 1D). 

### 2.2. Mitochondria are the Major Sites for Melatonin Synthesis in Oocytes

**Figure 2:**
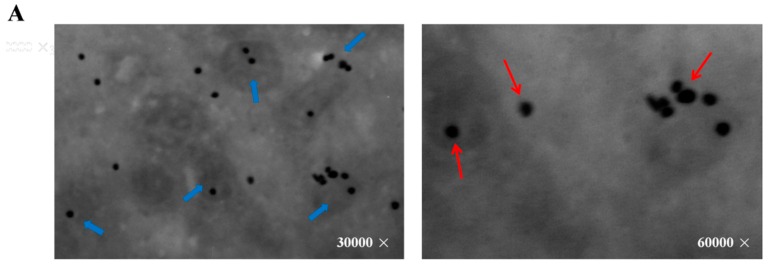

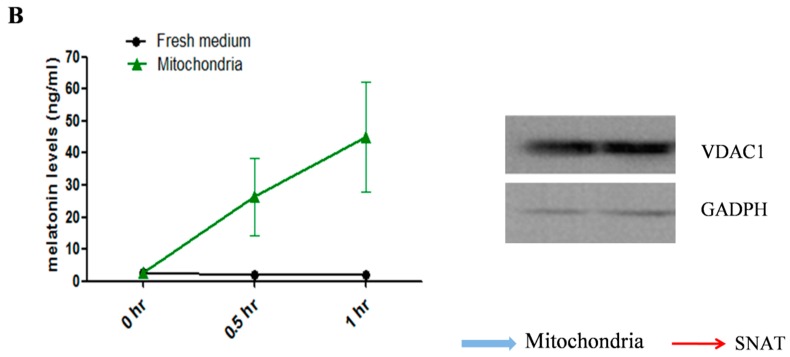
The localization of *SNAT* in oocyte and melatonin levels in mitochondria culture medium. (**A**) *SNAT* distribution sites. The red arrows point to *SNAT* enzyme (black dot), the blue arrows point to the mitochondria with *SNAT* localized in; (**B**) Melatonin concentrations in culture medium during mitochondria culture (mean ± SEM).

The image from Immuno-electron microscope (JEOL Ltd, Hitachi, Tokyo, Japan) showed that *SNAT* was distributed in mitochondria and cytoplasm (Figure 2A). To further test the ability of mitochondria in melatonin synthesis, we cultured mitochondria in the presence of the *SNAT* substrate serotonin. Results suggested that melatonin production was higher in serotonin-supplemented mitochondria than in the respective controls after 15 min (26.35 ± 11.980 *versus* 1.92 ± 1.985 ng/mL) and 30 min (44.96 ± 17.16 *versus* 1.93 ± 0.658 ng/mL) culture (Figure 2B). 

### 2.3. Melatonin Improves Oocytes Quality, and Accelerates IVF Embryo Development

**Figure 3:**
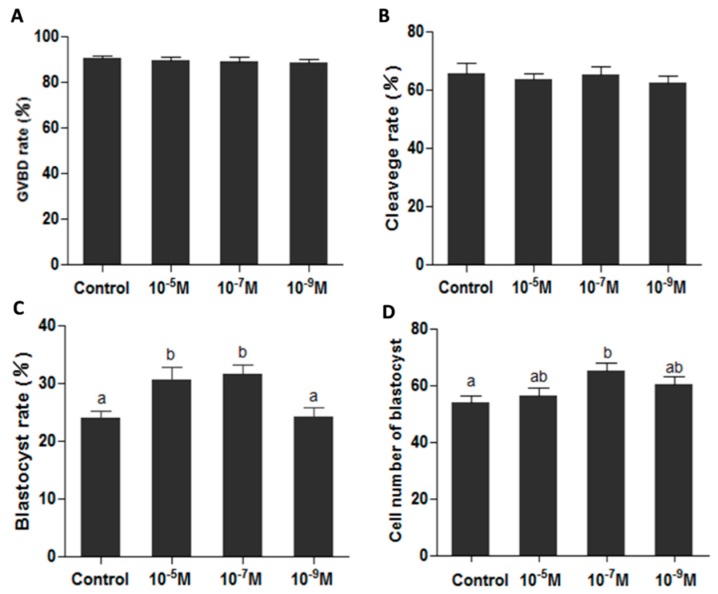
Effects of melatonin on oocyte’s maturation and subsequent IVF embryo development. (**A**) Effect of melatonin on GVBD rate (the percentage of GVBD oocytes); (**B**) Effects of melatonin on IVF embryonic development; (**C**) Effects of melatonin on blastocyst rate; (**D**) Effects of melatonin on the cell numbers of blastocysts (mean ± SEM of 1650 oocytes). The different superscript letters (a–b) represent a significant difference of these columns (*p* < 0.05).

Oocyte maturation medium added with melatonin exhibited a beneficial effect on oocyte’s maturation by detecting the developmental potential of IVF embryo in *in vitro* conditions. Analysis of an additional 1650 oocytes indicated that melatonin did not affect the germinal vesicle breakdown and cleavage rate (Figure 3A,B), while the blastocyst rates were significantly higher in melatonin-treated (10^−5^ and 10^−7^ M) groups than that in control group (0.31 ± 0.022 and 0.32 ± 0.016 *versus* 0.24 ± 0.013, *p* < 0.05) (Figure 3C), the cell numbers of blastocyst in melatonin-treated (10^−7^ M) group were also significantly higher (*p* < 0.05) (65.1 ± 2.55) than that in control group (53.9 ± 2.49) (Figure 3D).

### 2.4. Melatonin Ameliorates the Function of Mitochondria

**Figure 4:**
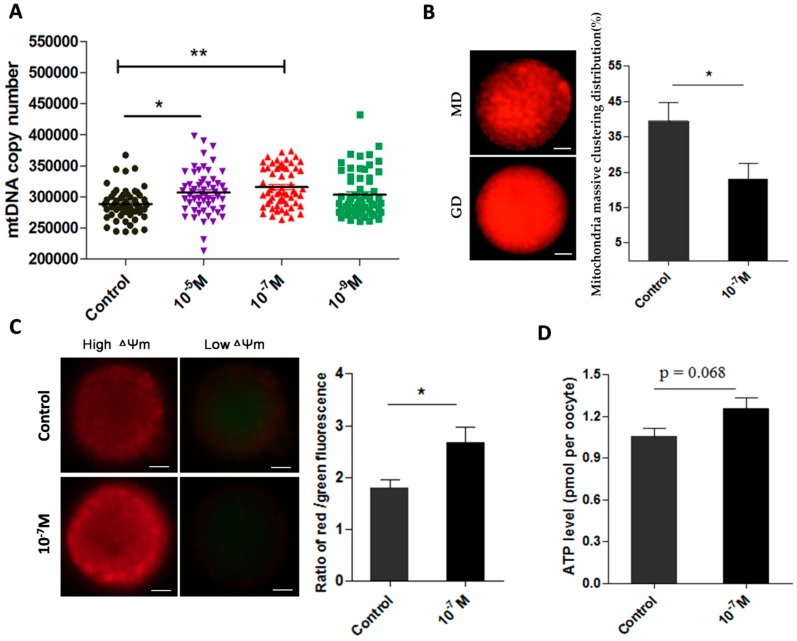
Effects of melatonin on mitochondria function and ATP production in MII-stage oocytes. (**A**) Cytoplasmic mtDNA copy number in MII-stage oocytes (mean ± SEM of 238 oocytes); (**B**) The state of mitochondria distribution (mean ± SEM of 178 oocytes), GD: granulated distribution; MD: massive clustering distribution. Scale bar = 20 μm; (**C**) Mitochondria membrane potential in MII-stage oocytes (mean ± SEM of 72 oocytes), Scale bar = 20 μm; (**D**) Cytoplasmic ATP levels in individual MII oocytes (mean ± SEM of 120 oocytes). “*” represent significant differences, *p* < 0.05; “**” represent significant differences, *p* < 0.01.

The data from 238 oocytes showed that melatonin treatments (10^−5^ and 10^−7^ M) significantly improved the mtDNA copy number of MII-stage oocyte (306,903 ± 4756 and 315,690 ± 4103 *versus* control 288,123 ± 3181, *p* < 0.05, *p* < 0.01, respectively) (Figure 4A). Analysis of 175 oocytes indicated that melatonin (10^−7^ M) decreased the massive clustering distribution rate of mitochondria in MII-stage oocyte (0.39 ± 0.050 *versus* 0.23 ± 0.045, *p* < 0.05) (Figure 4B). The ΔΨ_m_ in 10^−7^ M melatonin-treated group was significantly higher than that in control group (2.67 ± 0.288 *versus* 1.79 ± 0.166, *p* < 0.05) (Figure 4C). ATP level in melatonin-treated oocytes was also higher than that of controls (1.26 ± 0.079 *versus* 1.06 ± 0.057 pmol/per oocyte, *p* = 0.068) (Figure 4D).

### 2.5. Melatonin Promotes Meiotic Spindle Assembly

**Figure 5:**
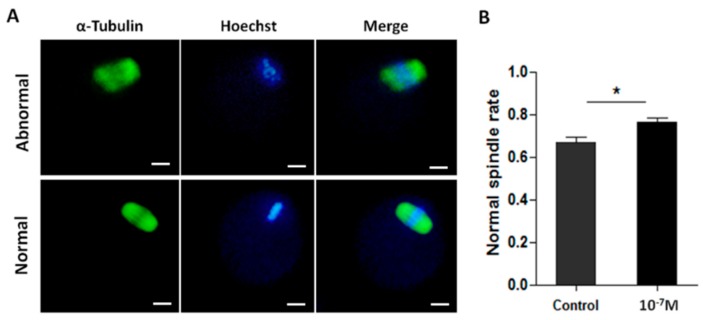
The effects of melatonin on meiotic spindle assembly of MII-stage oocyte. (**A**) Representative images of normal or abnormal meiotic spindle, Scale bar = 20 μm; (**B**) Incidence of normal spindle in melatonin-treated oocytes compared to the controls (mean ± SEM of 148 oocytes). “*” represent significant differences, *p* < 0.05.

Melatonin also significantly increased the normal spindle rate. The result from 148 oocytes treated with 10^−7^ M melatonin showed the normal rate of meiotic spindles was 76.2% ± 1.94% which was significantly higher than 67.0% ± 2.22% in control group (*p* < 0.05) (Figure 5).

### 2.6. Melatonin Protects Oocyte from Oxidative Damage

**Figure 6:**
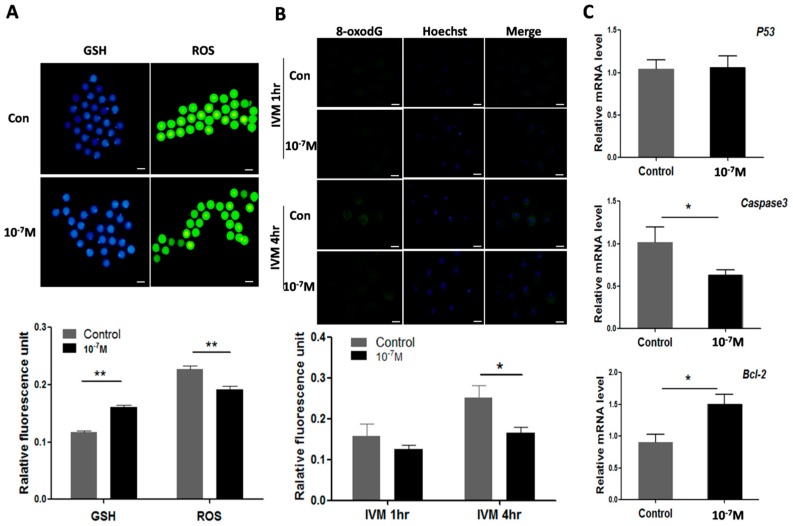
Effects of melatonin on ROS, GSH, 8-oxodG levels as well as apoptosis-associated genes expression in oocyte. (**A**) Effects of melatonin on levels of GSH and ROS in oocytes (mean ± SEM of 147oocytes), scale bar = 100 μm; (**B**) Effects of melatonin on 8-oxodG levels of oocyte during IVM (mean ± SEM of 198 oocytes), scale bar = 100 μm; (**C**) Effects of melatonin on the expression of apoptosis-associated genes. “*” represent significant differences, *p* < 0.05; “**” represent significant differences, *p* < 0.01.

The results showed that the levels of ROS were significantly lower in melatonin-treated oocyte (0.19 ± 0.006) than that of control (0.23 ± 0.007) (*p* < 0.01); In contrast, the levels of GSH were significantly higher in melatonin treated oocytes than those in controls (0.16 ± 0.003 *versus* 0.12 ± 0.002, *p* < 0.01) (Figure 6A). The 8-oxodG production was also reduced by melatonin treatment at 4 h of *in vitro* culture (0.25 ± 0.031 *versus* 0.17 ± 0.014, *p* < 0.05) (Figure 6B). Finally, the effects of melatonin on apoptosis-associated genes (*Bcl-2*, *Caspase3*, *p53*) were examined. The gene expression of *Bcl-2* was up-regulated by melatonin (1.50 ± 0.150) compared with control (0.91 ± 0.122) (*p* < 0.05). In contrast, the gene expression of *Caspase3* was down-regulated by melatonin treatment compared to the control (0.63 ± 0.059 *versus* 1.02 ± 0.181, *p* < 0.05). The expression of *p53*, however, was not influenced by melatonin (Figure 6C).

## 3. Discussion

In the current study, the mechanisms of melatonin on improving mice oocyte’s quality during maturation have been systematically investigated. Previous reports have indicated that melatonin may directly affect ovarian function to serve as a local regulator [31,32]. Melatonin was successively detected in follicular fluids of human [24], porcine [33] and bovine [34] ovaries. In addition, both *MT1* and *MT2* were identified in human granulosa cells [29]. Therefore, the high levels of melatonin in the follicle may constitute an important protective action for oocyte’s maturation. Although high concentrations of melatonin in ovarian follicular fluid have been reported, the original source of melatonin in the fluid is still understood completely. It was reported that, in rat, the highest melatonin levels occurred during the evenings of metestrus and diestrus, while the minimum levels were detected in the evening of estrus [35]. When [3H]-melatonin was injected into cats, this molecule was concentrated in the ovary (ten times higher than that in plasma) [36]. Similarly, in this study, we also observed that the melatonin level was higher in mice preovulation follicles than that in their contemporaneous serum (Figure 1C). Here, we identified that *SNAT* existed in oocytes during their maturation and the melatonin was also detected in the culture medium of oocytes (Figure 1D). These observations proved that oocytes had the capacity to synthesize melatonin, which was also demonstrated by Sakaguchi K *et al.* [30]. Based on the findings mentioned above, it appears that the high levels of melatonin in preovulatory follicles are mainly generated by the oocytes and the rest may be extracted from the blood. 

To further identify the subcellular site of melatonin synthesis in oocytes, mitochondria were targeted since mitochondria were hypothesized as the original sites of melatonin production [37]. Mitochondria are the main source of energy supply. They also function as a calcium sink as well as the regulators of cellular apoptosis. The mitochondrial dysfunction resulted in extensive ROS generation which is associated with a variety of pathological conditions and diseases [38,39]. Herein, the results indicated that the melatonin synthetic enzyme, *SNAT*, was localized in mitochondria as well as cytoplasm, and the isolated mitochondria retained the capacity to synthesize melatonin (Figure 2). Thus, we claimed that mitochondria are the sites for melatonin synthesis in oocytes during their maturation. In the current study, we failed to detect the classical mitochondria localization signal in the amino acid sequence of *SNAT* (data were not shown), thus, the mechanism by which *SNAT* was transported across mitochondria membrane is currently unknown. 

It appears that melatonin synthesized by mitochondria in oocytes plays a pivotal role in maintaining the normal physiology of oocytes. To prove it, melatonin was added to culture medium during *in vitro* oocyte maturation. Melatonin treatment improved the developmental potential of IVF embryo (Figure 3) which reflected an improved quality of oocytes. If these beneficial effects of melatonin on oocyte maturation occurred through its receptor is unknown, since the *MT1* and *MT2* were undetectable during oocyte maturation using immunofluorescence or RT-PCR (Figure 1A,B). It is also possible that the expression of both receptors is below detection threshold. These results were inconsistent with the observation in the study of bovine oocyte’s maturation [40]. This inconsistency may be species specific and, in order to resolve this issue, more studies are required.

Previous studies demonstrated that melatonin preserved the optimal mitochondrial function and homeostasis by reducing mitochondria oxidative stress [41,42,43]. In the current study, we identified that mitochondria *per se* synthesize melatonin. It is speculated that the melatonin level has a direct association with mitochondrial function. Indeed, melatonin supplementation significantly increased mtDNA copy number in MII-stage oocyte and influenced the degree of mitochondria granulated clustering (Figure 4A,B). Though the mitochondria distribution is a dynamic process [44], it is an important indicator of oocyte quality. A uniform, granulated distribution of active mitochondria in oocyte maturation and the early embryo specific period is necessary [45,46,47]. Melatonin also improved mitochondria membrane potential (ΔΨ_m_) of oocytes and enhanced their ATP production (Figure 4C,D). High ΔΨ_m_ is a precondition for ATP production [48]. In oocytes, both the mitochondria content and ATP levels are positively associated with the developmental competence of oocytes, that is, they promote the cytoplasmic maturation of oocytes and their IVF embryos’ development [49,50]. The discovery that mitochondria produce melatonin to ameliorate its function has important biological consequences. Mitochondria are the major source of ROS production and they need additional protection from oxidative stress. Melatonin is a potent, naturally occurring antioxidant. Its *de novo* production in mitochondria may provide the on-site protection.

The spindle abnormalities and chromosomal misalignment are increased during *in vitro* oocyte maturation, which might be a result of insufficient ATP production of cells cultured *in vitro* since the meiotic spindle assembly and maintenance require ATP [51]. Melatonin supplementation in IVM medium significantly reduced these abnormalities (Figure 5A). That melatonin increased ATP production (Figure 4) may contribute to oocyte spindle assembly. 

Another obstacle for oocyte maturation is over-produced ROS. The extensive ROS production jeopardizes the quality of oocytes and, therefore, hinders the oocyte’s maturation process and causes apoptosis. A relatively high level of ROS was detected in oocytes under their *in vitro* maturation (Figure 6A). To combat the negative effects of the excessive ROS and promote the oocyte’s maturation, antioxidants are frequently used in the *in vitro* culture system [52,53]. In this study, we found that melatonin addition to the culture medium significantly reduced the ROS and increased GSH level of the oocytes (Figure 6A). It is very possible that melatonin not only reduced ROS level via its direct ROS-scavenging action but also by improving mitochondrial function (Figure 4) which alleviates the ROS generation and increases the production of GSH [54]. In addition, melatonin was shown to reduce the levels of 8-oxodG which is a negative consequence of ROS (Figure 6B). As the major oxidative product of free nucleotide, 8-oxodGTP may be incorporated into DNA and cause mutation [55]. If the mutation is long-lasting, a negative effect on the oocyte quality and subsequent embryo development will be predicted. Furthermore, the apoptosis of oocytes may result from a poor mitochondrial function and excess ROS under *in vitro* conditions [56]. The apoptosis was triggered by *Caspase3*, which is activated by cytochrome c release from mitochondria. *Bcl-2* can interfere with cytochrome c release and therefore inhibit *Caspase3* activation [57,58]. In the current study, melatonin down-regulates *Caspase3* and up-regulates *Bcl-2* at the same time (Figure 6C), which protects oocyte from apoptosis. 

**Figure 7:**
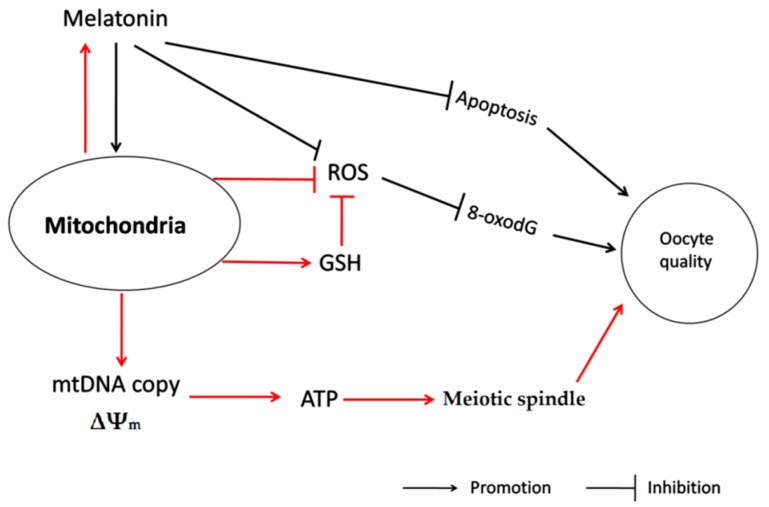
The action pathway connecting the beneficial effect of melatonin on oocyte quality during maturation.

In conclusion, this probably is the first study to identify the subcellular site of melatonin synthesis in oocytes. It was revealed that mitochondria are the organelles responsible for melatonin synthesis in oocytes. This discovery has significant biological implications. Melatonin *de novo* production in mitochondria may provide on-site protection. This was indicated by the improved mtDNA copy number, decreased clustering distribution of mitochondria, increased mitochondria membrane potential and enhanced ATP production in oocytes treated with melatonin. Therefore, it promotes meiotic spindle assembly and GSH production, reduces the ROS level, 8-oxodG and may inhibit oocyte apoptosis. These factors contribute to the beneficial effects of melatonin in improving the oocyte’s quality and, ultimately, the developmental ability of IVF embryo (Figure 7). The discoveries also provide a theoretical basis for application of melatonin in human test-tube baby technology.

## 4. Materials and Methods

### 4.1. Chemicals

Pregnant mare serum gonadotropin (PMSG) and human chorionic gonadotrophin (hCG) were obtained from Ningbo Hormone Products Co., Ltd. (Zhejiang, China). Melatonin, Sodium Pyruvate, BSA, Fetuin, Hyaluronidase, Penicillin and Streptomycin were purchased from Sigma-Aldrich (St. Louis, MO, USA). MEMa medium was purchased from Gibco (Carlsbad, CA, USA).

### 4.2. Animal Treatment

Three to five week old CD-1 mice (melatonin-proficient) were purchased from Vital River Laboratories Co., Ltd. (Beijing, China). Mice were housed under controlled conditions with temperature (22–26 °C) and a light/dark cycle 12/12 h. Animals were allowed to access to food and water *ad libitum*. PMSG (10 I.U.) was injected into mice abdomen in advance of 44–46 h to stimulate oocyte growth to sufficient size, and then hCG (10 I.U.) was injected to trigger oocyte maturation and ovulation. All animal studies have been approved by the animal care committees of China Agricultural University.

### 4.3. Analyses of Melatonin Receptors and Rate-Limiting Enzyme for Melatonin Synthesis

Mice were superovulated with an intraperitoneal injection of PMSG followed by hCG as mentioned above. Oocytes were collected at 0, 4 and 16 h respectively after hCG injection by puncturing the ovaries or oviducts with 1 mL disposable syringes. Retrieved oocytes were denuded with incubation in 100 IU/mL of hyaluronidase and then washed with PBS containing 0.5% BSA. The zona pellucida was removed via rapidly incubation in the acidified Tyrode’s solution (Irvine Scientific, Santa Ana, CA, USA). Thereafter, the oocytes were fixed and permeated in 4.0% neutral-buffered paraformaldehyde containing 0.3% Triton X-100 at 37 °C for 15 min. Nonspecific binding was blocked using PBS supplemented with 0.5% BSA, 0.1% Triton X-100, and 5% fatal bovine serum (FBS) at 37 °C for 1 h and then antibodies of *MT1*, *MT2* and *SNAT* (*MT1*, *MT2*, 1:100 dilution; *SNAT*: 1:300 dilution with PBS containing 0.5% BSA, and 5% FBS, Santa Cruz Bio Inc., Delaware Ave Santa Cruz, CA, USA) were added, respectively, and incubated at 4 °C for overnight. Oocytes were then washed three times with PBS containing 0.5% BSA and 0.01% Triton X-100 (15 min per wash) and incubated with donkey anti-goat IgG-FITC (1:100 dilution, Santa Cruz Bio Inc., Santa Cruz, CA, USA) goat anti-rabbit conjugated Alexa Fluor-568 (Life Technologies, Carlsbad, MA, USA) at 37 °C for 1 h. After washing, the cell nucleus was counterstained with Hoechst (Vector Laboratories) and analyzed by epifluorescence microscope (TE300; Nikon, Tokyo, Japan). 

### 4.4. Immuno-Electron Microscopic Identification of Melatonin Synthesizing Sites

PMSG (10 I.U.) was injected into mice abdomen in advance of 46 h to stimulate oocyte growing to sufficient size. The ovaries were collected from the abdomen cavity and they were fixed for 48 h in 2.5% glutaraldehyde. After washing, these ovaries were further dehydrated with a graded series of ethanol, xylene and, then, were embedded in epoxy resin. The follicles with the typical GV-stage oocytes were sectioned (100 nm) and placed on nickel grids and incubated in blocking solution (0.05% glycine in PBS) for 10 min. Then, the specimens were incubated with the *SNAT* primary antibody at 4 °C for 12–16 h and followed by incubated with anti-rabbit IgG (whole molecule)−gold-conjugated secondary antibody (1:20 dilution, Sigma-Aldrich, St. Louis, MO, USA) for another 2 h. After washing, these specimens were counterstained with 7% uranyl acetate, and washed again with distilled water. Images were acquired using a JEM-2200FS microscope (JEOL Ltd., Hitachi, Tokyo, Japan). 

### 4.5. Melatonin Assay with High Performance Liquid Chromatography (HPLC)

To measure the melatonin levels in ovary and serum during the course of oocyte maturation, mice were anesthetized with pentobarbital sodium by intraperitoneal injection at 4, 8 h after hCG injection. Blood was collected from caudal vein and centrifuged at 3000 r for 10 min to obtain the serum. The ovary samples of mice were weighed and homogenized immediately in 1 mL methyl alcohol and centrifuged at 12,000 rpm for 10 min at 4 °C. The supernatant was collected for melatonin assay. All procedures were performed under ice bath. To detecting the melatonin content in maturation medium, GV-stage oocytes were acquired by puncturing the ovary at 44–46 h after PMSG injection and denuded with hyaluronidase. For each group, 100 oocytes were cultured in MEMa medium supplemented with 10^−4^ M serotonin (Sigma-Aldrich St. Louis, MO, USA), PMSG (1 I.U./mL), hCG (2 I.U./mL), 0.3% BSA, Fetuin (1 mg/mL), Sodium Pyruvate (20 mM), Penicillin (75 μg/mL) and Streptomycin (50 μg/mL). The 100 µL of maturation medium was collected at 4 h, 8 h of the culture, respectively, for melatonin assay. All sample preparation and HPLC detection of melatonin were performed as previously reported [59]. The experiments were repeated at least 6 times.

### 4.6. Mitochondrial Isolation and Detection of Melatonin in Culture Medium

GV-stage oocytes were collected (See above), then divided into two groups (Melatonin-treated, Control) and cultured for 16 h in maturation medium. Then, mitochondrion were isolated using mitochondria isolation kit (Beyotime, Jiangsu, China) following the manufacturers’ instructions. The isolated mitochondria were then cultured in PBS supplemented with 10^−4^ M serotonin (the substrate of melatonin biosynthesis) at 37 °C for 15 and 30 min to test their melatonin-biosynthetic capacity. Melatonin in PBS was extracted and measured as mentioned previously. The experiments were independently repeated six times.

### 4.7. Western Blot Analysis

Following mitochondria isolation, its purity was verified by Western blot. VDAC1 and GADPH were selected as mitochondrial marker and cytoplasm marker, respectively. In brief, mitochondria samples were lysed in laemmli sample buffer (Bio-Rad, Hercules, CA, USA). Proteins were then separated by SDS-PAGE (12% acrylamide running gel) and transferred to a nitrocellulose membrane (BioTraceNT, Pall Corporation, Ann Arbor, MI, USA). Nonspecific binding to the membrane was blocked with 5% nonfat milk in Tris (10 mM), pH 7.5, NaCl (150 mM), and 0.1% Tween 20 at 37 °C for 1 h. Membranes were then incubated with primary antibodies (VDAC1 and GADPH (Abcam Inc, Cambridge, MA, USA) (1:4000 dilution, ab154856, ab128915, respectively)) at 4 °C, overnight, followed by incubation with horseradish peroxidase-conjugated secondary antibody (rabbit: 1:5000 dilution, ZB-2301, from ZSGB-Bio, Beijing, China) at 37 °C for another 1 h. Analysis was performed with enhanced chemiluminescence detection reagents (Applygen Technologies Inc., Beijing, China) and X-Omat BT film (HuaxingBio Inc., Beijing, China), according to the manufacturers’ instructions. 

### 4.8. Oocyte in Vitro Maturation (IVM), in Vitro Fertilization (IVF) and Embryo Culture 

GV-stage oocytes were collected by puncturing the ovary at 44–46 h after PMSG injection and then they were cultured in 60-μL of maturation medium containing 0, 10^−9^, 10^−7^, 10^−5^ M melatonin, respectively, at an incubator (5% CO_2_, 37 °C) for 16 h. After germinal vesicle breakdown (GVBD), the matured oocytes were denuded with hyaluronidase (Sigma-Aldrich, St. Louis, MO, USA). The percentage of GVBD oocytes (rate of GVBD) was recorded. Each cauda epididymidis was removed from adult male mice and the sperm were collected by gently squeezing the tissue with tweezers and placed into HTF medium (Sage Biopharma, Bedminster, NJ, USA) for capacitation at 37 °C for 1 h. The denuded oocytes were then incubated with sperms in HTF medium for 5 h. Pronuclei were washed to diminish the sperms and they were transferred into 60-μL KSOM medium (Millipore, Billerica, MA, USA) supplemented with 10% FBS for later development. Microscopic examination was then performed to monitor the development rates of both 2-cell and blastocyst. The cell numbers of blastocysts were assessed by Hoechst staining and the cells were mounted on a clean glass slide covering with a coverslip and examined using an epifluorescence microscope.

### 4.9. Detection of mtDNA Copy Number by qPCR 

The mitochondria-encoded NADH dehydrogenase subunit 5 gene (*MT-ND5*) was selected as an index to determine the mtDNA copy numbers. *MT-ND5* gene was amplified by PCR, then PCR products were extracted using the Tiangen gel extraction kit (Tiangen Biotech, Beijing, China), and cloned into the pEASYTM-T5 Zero Cloning vector (TransGen Biotech, Beijing, China) to obtain the recombinant plasmids. A single MII oocyte was added to a PCR tube with 5 µL of lysis buffer (50 mM Tris, 0.1 mM EDTA, 0.5% Tween-20 and 100 µg/mL Proteinase K), incubated at 55 °C for 30 min to lyse oocyte, and 95 °C for 10 min to inactivate Proteinase K. Then, amplification was performed by LightCycler 480 SYBR Green I Master Mix (Roche Diagnostics, Indianapolis, IN, USA) on LightCycler 480 II PCR machine (Roche Applied Science, Mannheim, Germany). Standard curves were determined using recombinant plasmids, which were serially diluted by 10^1^-10^5^ times. The real-time qPCR reactions consisted of SYBR Green (10 µL), forward and reverse primers (30 µM), template (5 µL) and ddH_2_O was added up to a total volume of 20 µL. The procedure was as follows: 95 °C for 10 min; 35 cycles of 95 °C for 10s and 60 °C for 10 s. For each sample, the mtDNA copy numbers are calculated using the threshold cycle number (*C*_t_) and corrected from the standard curve. *MT-ND5* primer sequences are listed in Table 1. The experiment was independently repeated 3 times.

### 4.10. Oocytes Mitochondrial Distribution Assay

MitoTracker Red CMRox (Life Technologies) was used to detect mitochondrial distribution. GV-stage oocytes were collected and divided into two groups (10^−7^ M Melatonin-treated and Control, respectively). Cells were cultured in maturation medium for 16 h. Then, MII-stage oocytes were collected and denuded from the adherence of cumulus cells and they were incubated in M2 (Sigma-Aldrich, St. Louis, MO, USA) medium supplemented with 100 nM dye at 37 °C for 40 min. Oocytes were then washed and analyzed by epifluorescence microscope (TE300; Nikon, Tokyo, Japan). Oocytes with a uniform granulated distribution of active mitochondria were scored as granulated distribution (GD), the oocytes with a massive clustering distribution of mitochondria were scored as massive distribution (MD). The images were observed and scored by three independent persons who are unaware this study. The experiment was independently repeated 3 times.

### 4.11. Detection of Mitochondrial Membrane Potential (ΔΨ_m_) and ATP Levels in Oocytes

The ΔΨ_m_ of the oocytes was measured using JC-1 (Beyotime, Haimen, Jiangsu, China). JC-1 fluorescence has two emission peaks, with red fluorescence indicating high mitochondrial membrane potential and green fluorescence indicating low mitochondrial membrane potential. Briefly, MII-stage oocytes in each group were exposed to 10 µg/mL of JC-1 at 37 °C for 15 min, and then washed with M2 medium to remove surface fluorescence. Fluorescence was observed using an epifluorescence microscope. The distribution of JC-1 dimers with red fluorescence and monomers with green fluorescence were detected using a red filter and green filter of the microscope, respectively. Fluorescence intensity was analyzed using ImageJ software (version 1.40; National Institutes of Health, Bethesda, MD, USA), the ratio of red to green fluorescence was used to analyze ΔΨ_m_. The experiments were independently repeated 3 times.

MII-stage oocytes were washed with PBS-PVA and a group of 10 oocytes were collected for ATP measurement, ATP levels were determined using a commercially available adenosine 5′-triphosphate (ATP) bioluminescent somatic cell assay kit (FLASC, Sigma-Aldrich, St. Louis, MO, USA) according to the manufacturer’s instructions. Briefly, oocytes were transferred into a 96-well plate with 45.8 µL ATP assay buffer, then 0.2 µL ATP probe, 2 µL ATP converter and 2 µL developer Mix were added. The plate was placed at room temperature for 30 min. ATP levels were measured using a luminometer (Bioluminat Junior, Berthold, Germany). The experiments were independently repeated 6 times.

### 4.12. Spindles Analysis

GV-stage oocytes were collected though puncturing the ovary which stimulated by PMSG, then divided into two groups (10^−7^ M Melatonin-treated, Control) and cultured for 16 h in maturation medium. MII-stage oocytes’ collection and immunofluorescence stain processed as described above. Mouse anti-α-tubulin was selected as the primary antibody (1:100 dilution, Santa Cruz Bio Inc., Santa Cruz, CA, USA), goat anti-mouse LgG-FITC was selected as the secondary antibody (1:100 dilution, Santa Cruz Bio Inc., CA, USA). For the spindle analyses, oocytes with barrel-shaped bipolar spindles having distinct and well-organized microtubule fibers, along with tightly aligned chromosomes on the metaphase plate, were scored as normal. The normal spindle rate is the percentage of oocytes with normal spindle. All experiments were independently repeated at least 3 times.

### 4.13. Measurement of Reactive Oxygen Species (ROS) and Glutathione (GSH)

The 2,7-dichlorodihydrofluorescein diacetate (H2DCFDA) (Beyotime, Jiangsu, China) and Cell Tracker Blue CMF2HC Molecular Probes (Invitrogen Inc., Carlsbad, CA, USA) were used to detect intracellular ROS and GSH level, respectively. GV-stage oocytes were collected through puncturing the ovary and divided into two groups (10^−7^ M Melatonin-treated, Control) and cultured for 16 h in maturation medium. Then, MII-stage oocytes were collected and denuded from adherence of cumulus cells, then they were incubated (in the dark) for 30 min in M2 medium containing H2DCFDA (10 µM) or Cell Tracker Blue (10 µM), respectively. The MII-stage oocytes were collected and denuded from the adherence of cumulus cells, and placed in 30 µL M2 droplets, and then the fluorescence was observed using an epifluorescence microscope. The fluorescence intensity was analyzed using ImageJ software (version 1.40; National Institutes of Health, Bethesda, MD, USA). The experiments were replicated 6 times.

### 4.14. 8-oxodG Assay

GV-stage oocytes were collected by puncturing the ovary, then divided into two groups (10^−7^ M Melatonin-treated, Control) and cultured in maturation medium. Oocytes were then collected and denuded from adherence of cumulus cells at 1, 4 h, respectively, after *in vitro* maturation and they were fixed in pure methanol at −20 °C for 20 min. Thereafter, they were incubated in PBS with 0.3% Triton x-100 for another 15 min. They were blocked with 5% FBS, 0.5% BSA in PBS at 37 °C for 1 h. Oocytes were then incubated with Alexa 488-conjugated avidin (10 μg/mL) (Invitrogen Inc., Carlsbad, CA, USA) in blocking solution at 37 °C for 1 h. After washing, DNA was counterstained with Hoechst and analyzed by epifluorescence microscope (TE300; Nikon). The fluorescence intensity of 8-oxodG was analyzed using ImageJ software. The experiments were replicated 6 times.

### 4.15. Gene Expression Assay with Reverse Transcriptional PCR or Real-Time qPCR

Oocytes were collected and denuded from the adherent cumulus cells at 0, 4 and 16 h, respectively, after hCG injection. The reaction procedure of RT-PCR was as follows: the initial denaturation at 95 °C for 5 min; denaturation at 95 °C for 30 s, annealing at 58 °C for 30 s and then extension at 72 °C for 30 s. The above procedures were repeated 35 cycles with a final extension at 72 °C for 5 min. For qPCR assay, GV-stage oocytes were collected and divided into two groups (10^−7^ M Melatonin-treated, Control) and cultured for 16 h in maturation medium. The amplification was performed by LightCycler 480 SYBR Green I Master Mix (Roche Diagnostics, Indianapolis, IN, USA) on LightCycler 480 II PCR machine (Roche Applied Science, Mannheim, Germany). The real-time qPCR reactions consisted of l SYBR Green (10 µL), forward and reverse primers (30 µM), template (2 µL) and ddH_2_O was added up to a total volume of 20 µL. The procedure was as follows: 95 °C for 10 min; 35 cycles of 95 °C for 10 s and 60–62 °C for 8–15 s; melting curve from 65 to 95 °C, increasing in an increment of 0.5 °C every 5 s. Normalization was performed using the housekeeping gene *Actin* as control. Primer sequences are listed in Table 1. The experiments were replicated at least 3 times.

### 4.16. Statistics Analysis

Data are expressed as mean ± S.E.M. Statistical analyses were used the univariate analysis of variance (ANOVA) with the aid of SPSS 19.0 statistical software followed by the Student *t*-test. *p* < 0.05 was considered statistically significant, and *p* < 0.01 was considered statistically highly significant.

## Figures and Tables

**Table 1:** Primers for RT-PCR and qRT-PCR.

Genes	Primer Sequence(5’–3’)	*T*_m_(°C)
*β-Actin*	Forward: CCAGCCTTCCTTCTTGGGTAT	60
	Reverse: AGGTCTTTACGGATGTCAACG	
*p53*	Forward: TGAGGTTCGTGTTTGTGCCTGC	60
	Reverse: CCATCAAGTGGTTTTTTCTTTTGC	
*Bcl-2*	Forward: ACCTGTGGTCCATCTGACCCTC	60
	Reverse: CCAGTTCACCCCATCCCTGA	
*Caspase-3*	Forward: CTGGAGAAATTCAAAGGACGGG	60
	Reverse: TGAGCATGGACACAATACACGG	
*MT-ND5*	Forward: ATAGCCTGGCAGACGAACAAGACA	60
	Reverse: AATTAGTAGGGCTCAGGCGTTGGT	
*MT1*	Forward: CCATTTCATCGTGCCTATG	58
	Reverse: GTAACTAGCCACGAACAGC	
*MT2*	Forward: TACATCAGCCTCGTCTGGCTCC	58
	Reverse: TTCCTCGTAGCCTTGGCCTTCC	
*SNAT*	Forward: TGAACATCAACTCCCTGAAACCT	60
	Reverse: TTCCCGCTCAATCTCAAACG	
